# Bilateral ovarian masses, massive ascites, and prior appendectomy: must it be ovarian cancer? ——a case report of pseudomyxoma peritonei 24 years after appendectomy

**DOI:** 10.3389/fonc.2026.1831067

**Published:** 2026-05-22

**Authors:** Sijie Feng, Shurui Yang, Kaixuan Yang, Xiuzhang Yu, Xiaoyu Niu

**Affiliations:** 1 Department of Gynaecology and Obstetrics, West China Second Hospital, Sichuan University, Chengdu, Sichuan, China; 2 Key Laboratory of Birth Defects and Related Diseases of Women and Children (Sichuan University), Ministry of Education, Chengdu, Sichuan, China; 3 Department of Pathology, West China Second Hospital, Sichuan University, Chengdu, Sichuan, China

**Keywords:** pseudomyxoma peritonei (PMP), ovarian cancer, Cytoreductive surgery (CRS), hyperthermic intraperitoneal chemotherapy (HIPEC), patient autonomy and rights

## Abstract

This case report presents a patient who underwent an appendectomy 24 years ago and was later admitted with bilateral ovarian masses and massive ascites. Ovarian cancer was suspected, and surgical intervention was prompted. During the operation, the abdominal cavity was found to be filled with yellow, jelly-like material. Both intraoperative frozen section and postoperative paraffin-embedded pathology confirmed that the ovarian masses were actually mucinous tumors originated from gastrointestinal duct and was clinically diagnosed as pseudomyxoma peritonei (PMP). This case highlights that even in patients with a long history of appendectomy and unremarkable gastrointestinal endoscopy, the possibility of ovarian metastasis from gastrointestinal mucinous tumors leading to PMP should not be overlooked. It also underscores the importance of long-term postoperative follow-up for PMP.

## 1 Introduction

Pseudomyxoma peritonei (PMP) is a distinct clinicopathological entity characterized by the diffuse accumulation of gelatinous fluid throughout the peritoneal surfaces and omentum (1). This disease is uncommon, with an estimated incidence of 2 cases per million population annually (2), though potentially higher at 3–4 cases per million due to its masquerading clinical presentation (3). The pathological classification of PMP has evolved over time. While the Ronnett system historically categorized PMP into three subtypes: Disseminated peritoneal adenomucinosis (DPAM), peritoneal mucinous carcinomatosis (PMCA), PMCA with intermediate or discordant features (PMCA-I/D) (4). However, to standardize terminology and better predict prognosis, the updated consensus classifies PMP into G1 (low-grade mucinous carcinoma peritonei), G2 (high-grade mucinous carcinoma peritonei), and G3 (high-grade mucinous carcinoma peritonei with signet ring cells). Notably, higher grades are consistently associated with a worse prognosis (5). The nonspecific clinical presentation, usually accompanied by appendiceal or ovarian masses, contributes to frequent misdiagnosis. In fact, the origin of PMP has been controversial since its initial description, but contemporary immunohistochemical (IHC) and molecular genetic analysis have definitively established the appendix as the principal site of origin, with the ovary representing a secondary yet much less common source, as PMP derived from ovarian mucinous tumors is exceedingly rare (6). As a matter of fact, when PMP occurs concurrently with both appendiceal and ovarian mucinous tumors in female patients, the appendix is identified as the primary source in the majority of cases (7). However, extreme metachronous presentations—particularly those occurring decades after an appendectomy—are exceedingly rare and can severely confound clinical diagnosis. The diagnostic challenge is further compounded when the clinical picture is entirely dominated by pseudo-gynecological symptoms. Herein, we present a complex case of a patient who developed PMP masquerading as advanced primary ovarian cancer 24 years post-appendectomy. By detailing this unusual clinical course, this report aims to provide clinical context on the extreme metachronous presentation of gastrointestinal-origin PMP, provide valuable insights into the biological behavior of this disease, and underscore the need for heightened vigilance into clinical management strategies.

## 2 Case report

**Table 1**.

**Table 1:** Timeline of the patient’s clinical history, diagnostic assessments, and therapeutic interventions.

Date/timeframe	Clinical event & intervention
2000 (24 years prior)	Underwent an open appendectomy.
2021 (3 years prior)	Underwent a laparoscopic cholecystectomy. Histological examination of a resected liver nodule confirmed mucinous adenocarcinoma (this critical medical history was withheld by the family until the current surgery).
April 2024	Onset of progressive abdominal distension, significant weight loss, and general abdominal discomfort.
November 6, 2024	Outpatient evaluation: Contrast-enhanced CT and endoscopy revealed massive bilateral multiloculated cystic adnexal masses, loculated ascites, and colonic polyps.
December 9, 2024	Hospital admission: Physical examination demonstrated a characteristic “frog-belly” appearance; transvaginal ultrasound was performed.
December 10, 2024	Preoperative assessment: Tumor markers (CA19-9, CA15-3, CEA) showed mild elevation. A preliminary diagnosis of suspected primary advanced ovarian cancer was established.
December 12, 2024	Exploratory Laparotomy: • Intraoperative frozen section indicated PMP of probable gastrointestinal origin. • The patient’s family disclosed the prior history of hepatic mucinous adenocarcinoma for the first time. • The surgical strategy was immediately adapted from a comprehensive gynecological staging procedure to targeted cytoreduction.
Postoperative	Final histopathological and immunohistochemical evaluations confirmed gastrointestinal-origin high-grade mucinous carcinoma peritonei. The patient was discharged and refused further surgical interventions or postoperative adjuvant therapies.

### 2.1 Case presentation

A 56-year-old female was admitted to our gynecology department on December 9, 2024, with her chief complaint of progressive abdominal distension over 8 months, accompanied by significant weight loss and abdominal discomfort. Her surgical history included an open appendectomy performed 24 years prior and a laparoscopic cholecystectomy 3 years before. Upon physical examination at admission, marked abdominal distension with a characteristic “frog-belly” appearance was observed, which precluded the palpation of the bilateral adnexa.

### 2.2 Diagnosis assessment

**Figure 1:**
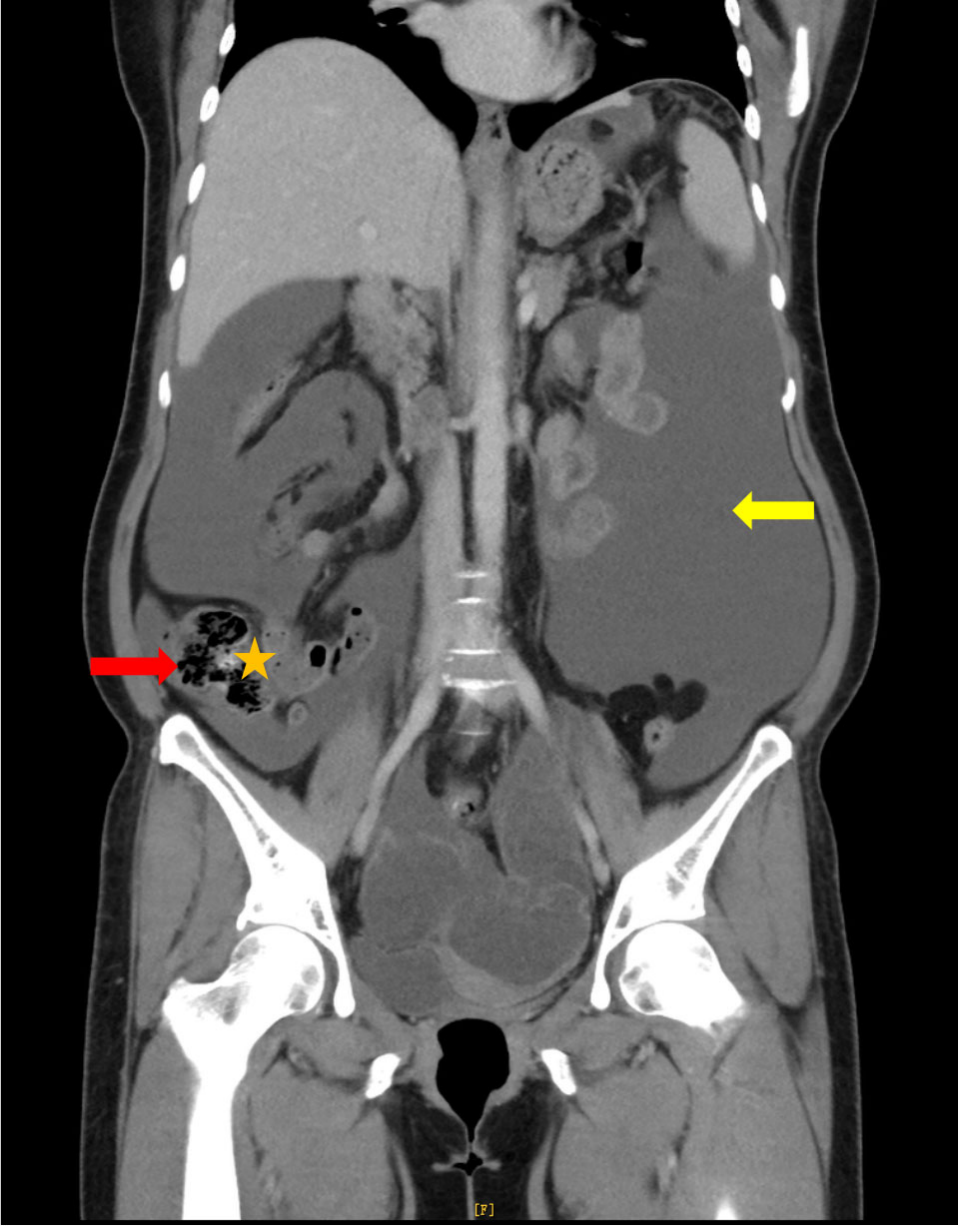
Preoperative contrast-enhanced CT of abdomen-pelvis-chest (coronal view). It demonstrates massive, partially loculated ascites filling the abdominal cavity (yellow arrow), alongside giant bilateral multiloculated cystic adnexal masses (red arrow) with punctate calcification on the cyst wall (orange star).

Prior to her admission (November 6, 2024), a contrast-enhanced computed tomography (CT) scan of the abdomen-pelvis-and chest (**Figure 1**) revealed massive, partially loculated ascites and diffuse peritoneal thickening with omental caking. Notably, bilateral multiloculated cystic adnexal masses were identified (Left: 8.2×7.8×13.9 cm; Right: 9.7×6.9×8.0 cm) with enhanced cyst walls, septations, and several punctate calcifications, “ovarian vascular pedicle” sign can be found bilaterally. Nodular implants can be seen across multiple compartments (including the splenic hilum, gastro-splenic space, gastric periphery, hepato-gastric space, and bilateral paracolic gutters). Multiple splenic cystic lesions can be seen, the largest cross-sectional measurement of which is 4.7×3.4 cm. On the same day, endoscopic evaluations identified a lateral spreading tumor (LST) in the transverse colon, multiple sigmoid polyps, and chronic atrophic gastritis (C1, active) with *H. pylori* infection. Upon inpatient admission (December 9, 2024), a transvaginal ultrasound further characterized the massive bilateral complex cystic adnexal masses, exhibiting irregular morphology, poorly defined borders, and an absence of significant vascularity. Subsequent tumor marker profiling on December 10, 2024, revealed mildly elevated levels: CA19-9: 34.6 U/mL, CA15-3: 22.4 U/mL, and CEA: 34.8 ng/mL.

### 2.3 Therapeutic intervention and intraoperative findings

**Figure 2:**
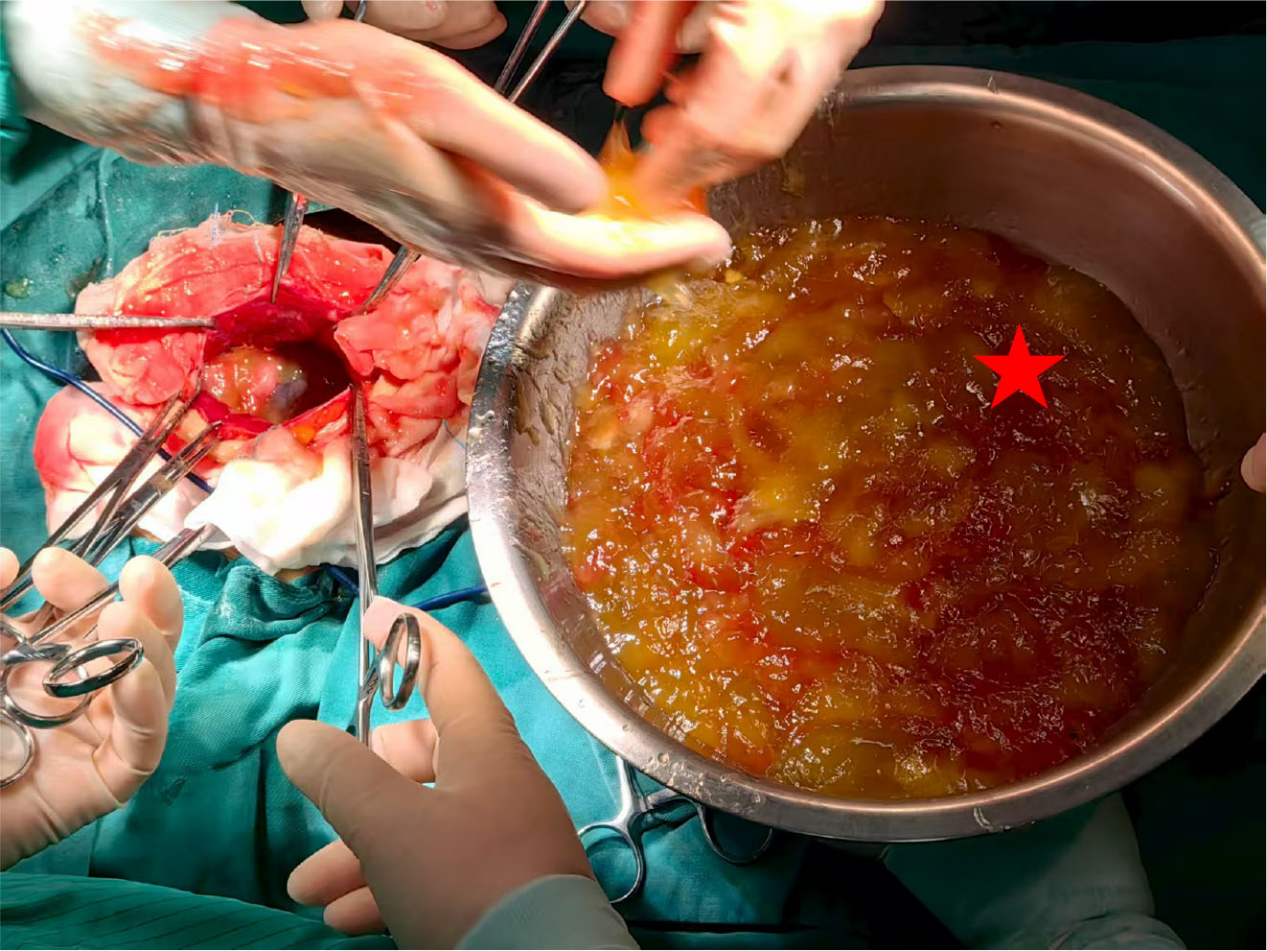
Intraoperative macroscopic findings. The exploratory laparotomy revealed the abdominal cavity filled with yellow, gelatinous mucinous ascites (red star).

**Figure 3:**
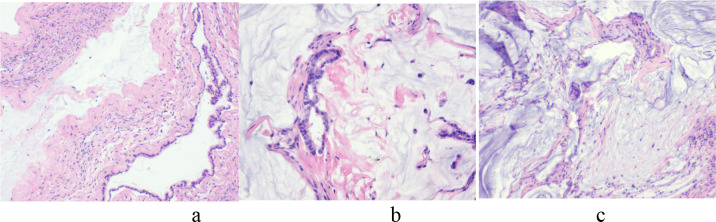
Intraoperative frozen section histopathology demonstrating mucinous tumor involvement across multiple abdominopelvic regions (200× magnification). **(a)** mass from the right adnexa showing abundant extracellular mucin pools. **(b)** nodules from the abdominal wall containing atypical mucinous epithelial cells. **(c)** nodules from the gastric serosa exhibiting similar mucinous neoplastic epithelium.

According to all the preoperative examination, preliminary diagnosis “Pelvic mass (ovarian carcinoma suspected)” was made. Consequently, an exploratory laparotomy was performed on December 12, 2024. Intraoperative exploration initially revealed that the abdominal cavity was completely filled with yellow gelatinous material, approximately 6500g of which was evacuated (**Figure 2**). The peritoneal surfaces demonstrated widespread involvement, with numerous nodular implants measuring 0.3-2.0 cm diffusely distributed across the entire parietal peritoneum and bilateral paracolic gutters, and a characteristically thickened, “caked” omentum can be observed. The uterus and bilateral adnexa were studded with similar tumor nodules ranging from 0.5-2.5 cm in size. Both ovaries were markedly enlarged - the left presenting as an irregular 10×9×9 cm cystic mass and the right as an oval 9×8×7 cm cystic lesion, both exhibiting multiloculated structure with intact capsules and containing yellow gelatinous fluid. Frozen section analysis of the right adnexal mass identified a mucinous neoplasm featuring abundant mucin lakes and epithelial cells with mild atypia, most consistent with a low-grade mucinous tumor of probable gastrointestinal origin (**Figure 3**). Therefore, gastrointestinal surgeons were invited for intraoperative surgical consultation. Further exploration of the upper abdomen revealed marked omental thickening with a “cake-like” appearance and multiple dense adhesions to surrounding structures. Numerous friable, nodular lesions (ranging from 0.5 to 3 cm in diameter) were diffusely scattered across the omentum, colonic mesentery, diaphragmatic surface, liver, gastric serosa, pancreas, spleen, and intestinal walls. A distinct 4 cm mass was palpated at the upper pole of the spleen. Notably, neither the gallbladder nor identifiable appendiceal remnants were visualized. Representative nodules from the abdominal wall and gastric serosa were submitted for frozen section analysis (**Figure 3**), the preliminary pathological assessment identified suspicious clusters of free-floating mucinous tumor epithelium. Regarding to macroscopic finding and rapid frozen section results, a preliminary intraoperative diagnosis of PMP of probable gastrointestinal origin was established. To ensure diagnostic accuracy, a detailed re-inquiry onto the patient’s clinical history was conducted with her family, they disclosed for the first time that during the cholecystectomy performed three years ago, concurrent resection of hepatic surface nodules had been performed, the final pathology at that time had revealed chronic cholecystitis, while the hepatic nodules were diagnosed as mucinous adenocarcinoma. The family had made the decision to withhold this news of the malignant diagnosis from the patient, subsequently decline recommended adjuvant therapies such as intraperitoneal chemotherapy. The patient had only received sorafenib therapy for six months, with no regular follow-up thereafter. After thorough communication with the family regarding the current findings and prognosis, the agreement has been reached to proceed with bilateral salpingo-oophorectomy, omentectomy, and splenectomy. Aggressive cytoreduction was attempted, with either resection or cauterization of all visible metastatic deposits throughout the peritoneal cavity. However, complete resection was not achievable, with residual tumor nodules exceeding 1 cm in maximum dimension remaining.

### 2.4 Postoperative pathological evaluation

**Figure 4:**
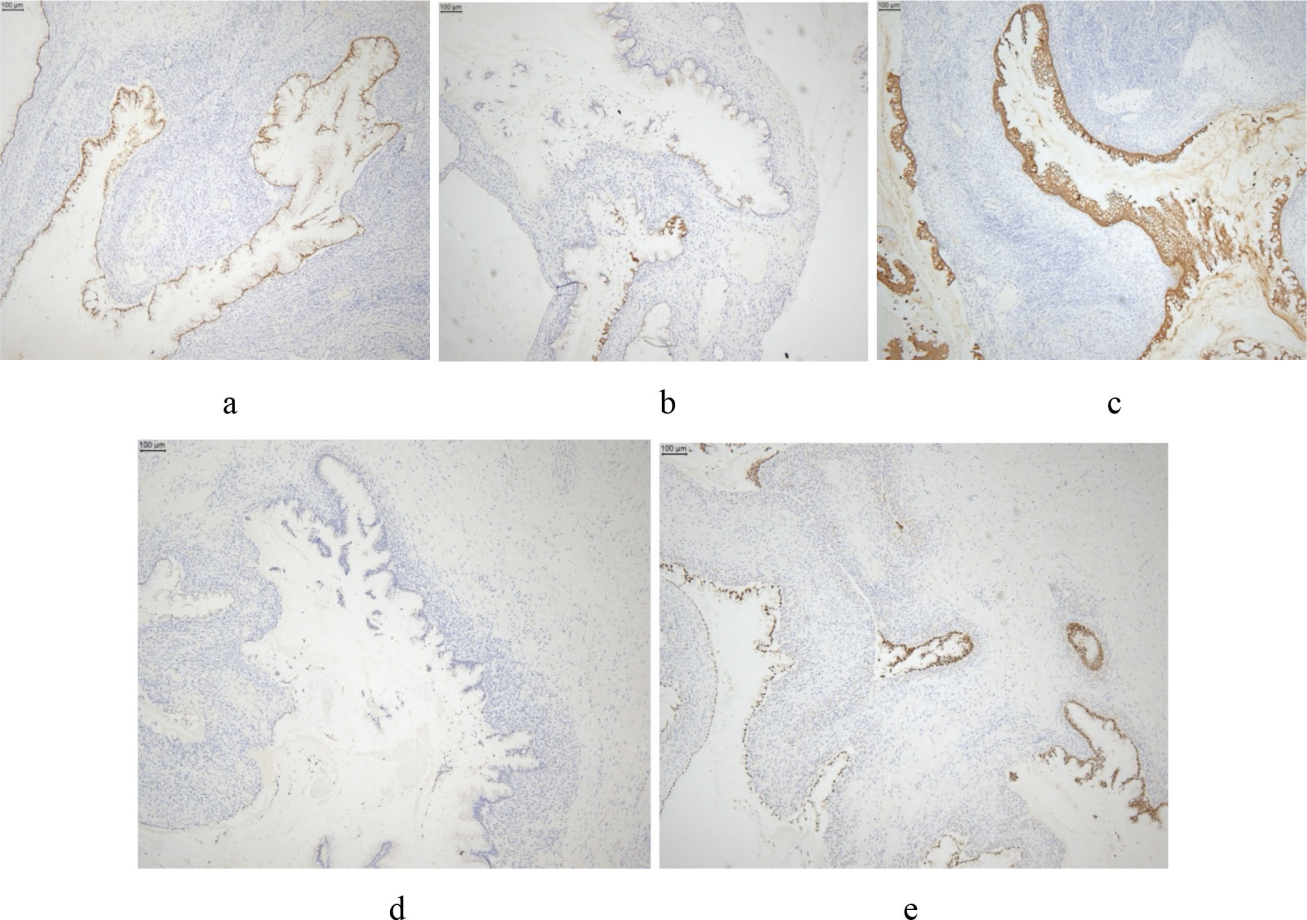
Postoperative immunohistochemistry (IHC) analysis of the left adnexal mass (40× magnification). The tumor cells exhibit a characteristic gastrointestinal immunophenotype, showing **(a)** strong positive nuclear staining for CDX2, **(c)** diffuse positive cytoplasmic staining for CK20, and **(e)** positive nuclear staining for SATB2. Conversely, the tumor shows **(b)** only focal positive staining for CK7 and **(d)** completely negative for the gynecological marker PAX8.

The postoperative pathological examination demonstrated extensive involvement by mucinous tumor across multiple sites including bilateral adnexa, peritoneal nodules, splenic capsule, and omentum, all characterized by abundant mucin lake formation and epithelial cells exhibiting mild-to-moderate atypia. IHC analysis revealed a distinct profile: CK20(+), CDX2(+), SATB2(+) with focal CK7 and p16 expression, wild-type p53, intact DPC4, and low Ki-67 proliferation index (approximately 5%), while being uniformly negative for ER, PR, PAX-8, GATA-3, and WT-1 (**Figure 4**). This comprehensive IHC pattern, definitively established a gastrointestinal primary origin and confirmed at least a high-grade mucinous neoplasm with widespread peritoneal dissemination. Subsequent histopathological consultation revealed metastatic well-differentiated mucinous adenocarcinoma involving bilateral adnexa (surfaces and parenchyma of both ovaries, serosal surfaces and smooth muscle interstitium of both fallopian tubes), spleen, omentum, and peritoneal nodules. Based on the IHC results from the left adnexal specimen, the result is the same as tumor profile showed before, additional recommended IHC stains were done to support a gastrointestinal primary include: Villin+++, CEA++, and Cadherin-17+++. While neither morphology nor IHC support gynecological origin. Integrating the widespread peritoneal dissemination, distinctive morphological features, and the IHC profile, the final diagnosis was high-grade mucinous adenocarcinoma peritonei (G2) of gastrointestinal origin, presenting with extensive metastases to the bilateral adnexa, spleen, and omentum. Both morphological and IHC evidence definitively excluded a primary gynecological malignancy.

**Figure 5:**
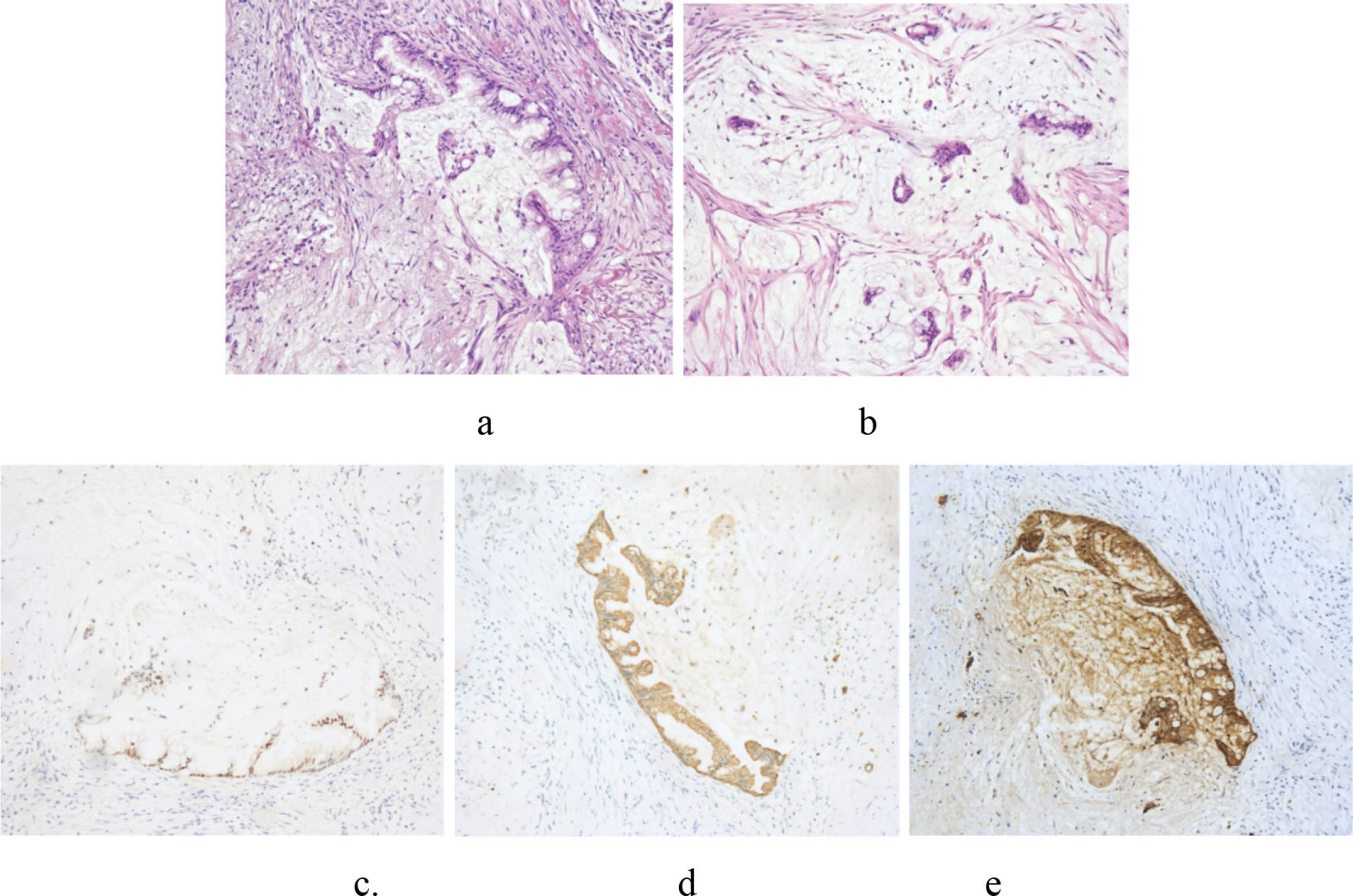
Re-evaluation of the pathological sections from the hepatic nodule resected 3 years prior (200× magnification). **(a, b)** hematoxylin and eosin (H&E) staining shows mildly atypical, hypersecretory mucinous glandular epithelium within extensive mucin aggregates. Immunohistochemical staining demonstrates **(c)** positive nuclear expression of CDX2, **(d)** strong cytoplasmic expression of CK20, and **(e)** positive apical membranous expression of villin, strongly supporting a gastrointestinal primary origin.

Subsequent retrieval and review of the surgical records from the cholecystectomy performed three years ago revealed significant findings. The operation record showed that documented 100 mL of dark-colored ascitic fluid and diffuse grey-white nodules involved both the hepatic surface and subdiaphragmatic region. Pathological examination of the resected liver nodules had confirmed mucinous adenocarcinoma, with IHC staining showing CK7(+), CK20(+), CDX2(+), and Villin (+) expression, while being negative for PAX8, CA125, and TTF1, which is a profile consistent with gastrointestinal origin. Upon re-evaluation of the pathological sections, we observed positive expression of CDX2, CK20, Villin (**Figure 5**), furthermore, no identifiable hepatic parenchyma was observed within the liver nodules.

### 2.5 Postoperative follow-up

The patient recovered uneventfully and was discharged seven days later. At the one-year postoperative follow-up conducted via telephone, the patient reported no adjuvant therapy since her discharge. She maintained an excellent performance status, with normal appetite and sleep patterns. The patient was asymptomatic, specifically denying abdominal distension or other discomfort. Laboratory investigations showed no significant abnormalities. Despite our recommendations, the patient declined further medical or surgical interventions.

## 3 Discussion and literature review

### 3.1 Clinical significance and extreme metachronous presentation

PMP is a distinct clinicopathological entity characterized by the abundant production of mucinous material by mucinous neoplastic epithelial cells, resulting in the accumulation of yellow gelatinous ascitic fluid within the peritoneal cavity (8). This condition typically develops following rupture of mucin-producing tumors originating from abdominopelvic organs, leading to peritoneal dissemination and implantation on omentum, visceral and parietal peritoneal surfaces (9). Analysis of Dutch nationwide data identified the appendix as the primary site of origin in 86.4% of PMP cases, with low-grade appendiceal mucinous neoplasms (LAMN) comprising 68% of this patient subgroup. This predilection for appendiceal origin stems from its unique histology and anatomy, the abundant glandular mucosa combined with narrow lumen of the appendix facilitate both excessive mucin production and propensity for obstruction hence rupture. It is distinct from the metastatic patterns of mucinous tumors arising from other sites like colon which typically spread via lymphatic or vascular routes. Although peritoneal implantation is the most common metastatic route for ovarian mucinous tumors, the presence of the dense ovarian surface capsule—the tunica albuginea—and the fact that spread typically requires malignant transformation restrict the overall incidence of peritoneal seeding from a primary ovarian origin. Additionally, true ovarian-origin PMP generally arises in the context of mucinous tumors developing within mature cystic teratomas (10). Notably, even when preoperative imaging and intraoperative findings suggest other organs as potential primary sites, the appendix remains the most probable source in most cases. This underscores the critical importance of meticulous appendiceal examination during surgical exploration for definitive diagnosis and management. Current guidelines recommend appendectomy as an essential and important component of surgical treatment for PMP regardless of whether gross abnormalities are visible intraoperatively (6).

This case raises an important unresolved question: how could a gastrointestinal-origin PMP develop in a patient who underwent appendectomy 24 years prior and showed no abnormalities on gastrointestinal endoscopy? While the presence of hepatic nodules discovered during the previous surgery might suggest a liver origin, this hypothesis was ruled out. A thorough re-evaluation of the pathological slides from the hepatic nodules failed to identify any functional hepatic parenchyma. This finding not only excludes a primary liver malignancy but also confirms the persistence of the lesion for at least three years. According to the principal of PMP originated from the appendix and its high morbidity, we finally postulate that a mucinous tumor may have already been present at the initial appendectomy but remained undetected, either because it had not yet ruptured or because the appendix wasn’t properly examined during surgery. It also serves as a clinical testament to the paradoxical behavior of mucinous neoplasms: they are characterized by low-grade malignancy coupled with a high propensity for recurrence, and it can exhibit extremely indolent biological behavior, remaining dormant for decades before presenting with explosive dissemination and masquerading as primary ovarian cancer or disease from other system, even the high-grade mucinous adenocarcinoma identified in this case exhibited such a prolonged clinical course, hence can recur even after seemingly complete resection. Studies show that 24.2% of PMP patients experience recurrence after CRS/HIPEC, with 20.7% recurring within five years (11), underscoring the critical importance of diligent postoperative follow-up. Even when intraoperative and pathological findings suggest a gynecologic origin, patients should undergo regular tumor marker tests and abdominopelvic CT scans to monitor for appendiceal or other gastrointestinal lesions. For patients with confirmed gastrointestinal-origin PMP who retained their ovaries during surgery, ongoing surveillance is also essential to detect potential ovarian involvement. Compared to previously published literature, the present case is remarkably unique due to its extraordinary 24-year latency period and pseudo-gynecological presentation. While PMP recurrence or delayed presentation does happen, it typically occurs within 5 years following initial appendiceal disease. Recent large-scale retrospective cohorts have established that the median disease-free interval for PMP following surgical resection typically ranges from 19 to 21 months (12, 13), and the maximum interval between appendectomy and first PMP diagnosis documented to date was 10 years (14), so it is recommend that follow-up examinations should be done for at least 10 years for a long time. However, as for this case, the postoperative follow-up period should be prolonged.

Even more astonishingly, researches have confirmed the highly invasive nature of G2 or G3 lesions, which consistently portend significantly worse survival outcomes (8, 15), but this patient experienced a 3-year period of clinical stability following an incidental intraoperative discovery of high-grade hepatic mucinous metastasis, let alone receiving no targeted systemic chemotherapy. This pronounced disconnect—between an aggressive high-grade histopathology and an exceptionally indolent, decades-long clinical dormancy—is exceedingly rare. It challenges the conventional understanding of high-grade PMP progression, suggesting that these tumors can harbor poorly understood mechanisms of prolonged dormancy before transitioning into explosive dissemination.

### 3.2 Diagnostic dilemmas

Female patients presenting with nonspecific symptoms such as abdominal distension, pain, appetite loss, or even bowel obstruction, coupled with imaging findings of adnexal cystic masses and significant ascites, gynecologists usually give an initial diagnosis of ovarian malignancy. It is crucial to note, however, that PMP—a disease process defined by peritoneal dissemination of mucinous tumor, most commonly from the appendix or gastrointestinal tract—must be considered in the differential diagnosis due to its overlapping clinical presentation. However, this case exemplifies the diagnostic dilemma: despite negative gastrointestinal endoscopic findings and a remote appendectomy history (which would ostensibly exclude gastrointestinal origins), both intraoperative exploration and final pathological confirmation unexpectedly identified a PMP resulting from primary gastrointestinal tumor. This phenomenon underscores the remarkable masquerading capability of PMP. Inadequate preoperative evaluation or insufficient understanding of this disease frequently may lead misdiagnosis, it may cause severe therapeutic consequences since the management paradigms for ovarian malignancies and gastrointestinal mucinous tumor-derived PMP diverge fundamentally in both surgical scope and adjuvant therapy.

From a treatment perspective, for early-stage (FIGO I) ovarian cancer, comprehensive surgical staging is standard, encompassing total hysterectomy with bilateral salpingo-oophorectomy, omentectomy, and pelvic lymph node dissection. In advanced cases (FIGO II-IV), primary cytoreductive surgery aims to maximally debulk all visible disease, including primary and metastatic lesions. When chemotherapy is indicated for ovarian cancer, platinum-based regimens (cisplatin/carboplatin) typically form the cornerstone of systemic treatment (16).In contrast, no matter the origin, PMP management principally focus on appendectomy, with intraoperative decisions regarding bilateral oophorectomy and omentectomy and organs removal based on disease involvement, often without routine lymphadenectomy. When widespread peritoneal dissemination precludes complete resection, CRS is pursued, if LAMN with transmural invasion or HAMN is examined, HIPEC utilizing agents such as mitomycin C or oxaliplatin will be strongly recommended (17, 18). Therefore, misdiagnosis leading remaining of appendix significantly increases possibility of recurrence. Moreover, for female patients who are longing to give birth, unnecessary oophorectomy and hysterectomy not only raise risks of the surgery but also induce premature menopause and irreversible loss of fertility. Additionally, if retroperitoneal lymph node dissection is performed during surgery, opening the retroperitoneal space may facilitate the spread of mucinous tumors, thereby potentially increasing the risk of metastasis in this area.

Therefore, it is critical to conduct a meticulous differential diagnosis. The implementation of a standardized diagnostic pathway is essential to reach correct diagnosis. The evaluation should begin with comprehensive history-taking, with particular attention to prior gastrointestinal surgeries, followed by detailed physical examination where gelatinous ascites may manifest as distinctive “dough-like” consistency on palpation. Preoperative contrast-enhanced CT imaging of the abdomen and pelvis provides critical differentiation: ovarian malignancies typically demonstrate nodular peritoneal implants with low-density ascites, whereas PMP characteristically presents with mucinous masses accompanied by “scalloping indentation” of solid organs (liver/spleen) and higher-density, gelatinous ascites displaying a “fan-shaped” mesenteric distribution. Tumor marker profiling further aids discrimination: while ovarian malignancies predominantly elevate CA125, gastrointestinal mucinous tumor-resulted PMP more commonly shows elevated CEA, CA19-9, and EpCAM levels (19). For patients with PMP who deemed eligible for CRS+HIPEC, preoperative gastrointestinal endoscopy remains mandatory to exclude synchronous malignancies (20). In this case, the management plan, following the endoscopic diagnosis of a LST in the transverse colon, should entail endoscopic mucosal resection (EMR) performed during a subsequent admission to the Internal Medicine service, after the completion of the scheduled gynecological surgery. Intraoperatively, concurrent colectomy or local resection was also deferred after a comprehensive assessment of the surgical risks. Nonetheless, it is undeniable that a definitive characterization of the transverse colon LST remains elusive: it could represent a synchronous, independent adenoma or adenocarcinoma, or alternatively, a rare implantation of the mucinous tumor mimicking an LST. Therefore, a standardized preoperative evaluation for PMP patients must include complete gastrointestinal endoscopy with biopsy of any suspicious lesions. Should this patient require additional cytoreductive surgery (CRS) in the future, meticulous intraoperative examination of the transverse colon must be accorded high priority.

However, ancillary tests alone cannot serve as definitive diagnostic criteria. This case exemplifies such limitations: despite elevated CEA levels, the abdominopelvic CT demonstrated only scattered nodular lesions without pathognomonic findings like “fan-shaped” mesenteric involvement or organ scalloping indentation. Consequently, histopathological confirmation becomes imperative, rendering intraoperative frozen section analysis critical for determining both surgical approach and extent of resection. In this particular case, frozen section evaluation of the right adnexal mass revealed a low-grade mucinous neoplasm of probable gastrointestinal origin. This pivotal finding prompted immediate intraoperative surgical oncology consultation, leading to a tailored multidisciplinary procedure comprising peritoneal nodule resection, omentectomy, and splenectomy. Notably, this approach avoided unnecessary hysterectomy and lymphadenectomy, thereby circumventing associated risks.

Beyond the pivotal influence of intraoperative frozen section analysis on surgical decision-making, comprehensive postoperative histopathological examination remains equally indispensable. Current guidelines emphasize that all appendiceal neoplasms and PMP cases require definitive histological review by pathologists with specialized expertise in peritoneal surface malignancies (20). This case exemplifies the diagnostic evolution that may occur between intraoperative and final pathology assessments: while frozen section of the right adnexal mass initially suggested a at least low-grade mucinous tumor of probable gastrointestinal origin, the postoperative histopathological examination sections following multidisciplinary review ultimately confirmed it as metastatic well-differentiated mucinous adenocarcinoma with widespread peritoneal involvement, but definitively excluding gynecological origin through IHC profiling. Therefore, the patient in this case should be followed up actively after surgery to assess following treatment such as right hemicolectomy and CRS+HIPEC.

### 3.3 Treatment strategy

The treatment paradigm for PMP requires careful stratification based on histopathological findings and disease extent. For LAMN without evidence of appendiceal perforation intraoperatively, appendectomy alone may suffice. However, when perforation is identified with concomitant peritoneal cellular mucin deposition, aggressive cytoreductive surgery (CRS) combined with hyperthermic intraperitoneal chemotherapy (HIPEC) becomes imperative. In cases of high-grade appendiceal mucinous neoplasms (HAMN) or more advanced lesions, the surgical approach should be expanded to right hemicolectomy alongside CRS+HIPEC. Contemporary outcomes from high-volume centers demonstrate that optimal CRS (defined as residual tumor <2.5 mm) followed by HIPEC yields superior disease-free survival (DFS) and overall survival (OS) rates (21). The satisfactory synergistic effect of CRS+HIPEC has established them as the gold-standard therapeutic approach for PMP. Moreover, the ovaries represent both a potential primary site outside the appendix for mucinous neoplasms and the most frequent location of metastatic involvement in PMP, meanwhile, ovarian metastasis independently correlates with poorer prognosis even when complete cytoreduction is achieved and histological grade is at the same level (22). This prognostic significance has led specific management recommendations: bilateral oophorectomy should be routinely performed in postmenopausal women regardless of apparent ovarian involvement, while ovarian preservation in premenopausal patients requires judicious consideration of tumor biology and reproductive priorities. In the present case, the ovarian-dominant presentation necessitated bilateral salpingo-oophorectomy as part of the therapeutic strategy. Additionally, recent evidence suggests that molecularly targeted therapies may be a potential efficacy of precision oncology approaches in PMP (23). Preoperative intravenous chemotherapy was also used to treat PMP before, but studies have shown that these neoadjuvant approaches may be unhelpful or paradoxically worsen DFS and OS outcomes in high-grade appendiceal (HGA) or high-grade colorectal origins.

### 3.4 Strengths and limitations of the clinical approach

To further refine clinical management standards, it is essential to summarize the diagnostic and therapeutic approach utilized in this case. A primary strength of our management was the prompt utilization of intraoperative frozen section analysis combined with the immediate integration of a multidisciplinary surgical team. Once the frozen section suggested a gastrointestinal origin, the surgical strategy was rapidly adapted. This crucial intraoperative pivot successfully prevented an inappropriate and potentially morbid comprehensive gynecological staging procedure. However, several notable limitations must be acknowledged. First, owing to the extreme delay in diagnosis and the massive disease burden at presentation, complete cytoreductive surgery was not archived. Second, the preoperative evaluation was severely hindered by incomplete anamnestic data. Had the patient’s family disclosed the histological confirmation of hepatic mucinous adenocarcinoma from three years prior, a gastrointestinal primary would have been highly suspected preoperatively. This would have fundamentally altered the initial surgical planning, potentially allowing for a scheduled, single-stage definitive CRS and HIPEC procedure. Finally, the patient’s refusal to pursue postoperative adjuvant therapies or further surgical interventions significantly compromises the potential for long-term disease control.

### 3.5 Patient autonomy and ethical considerations

This case presents significant ethical challenges regarding patient consent and family involvement in medical decision-making. Three years prior, the patient’s hepatic nodule resection during cholecystectomy revealed mucinous adenocarcinoma, yet the family deliberately withheld this diagnosis, declined recommended chemotherapy, and avoided follow-up monitoring. While we cannot definitively judge whether these decisions directly contributed to the current PMP development, this situation highlights the fundamental tension between family-protective intentions and core medical ethics principles. The World Medical Association’s Declaration of Helsinki explicitly states: “In medical research involving human subjects capable of giving informed consent, each potential subject must be adequately informed of the aims, methods … and the voluntary nature of participation.” Although family consultation remains culturally appropriate in many contexts, ultimate decision-making authority should reside with competent patients. Clinicians must carefully balance cultural sensitivities with ethical obligations, when full disclosure to patients seems contraindicated, thorough discussions with family members become imperative. Critical outcome data underscore this necessity: untreated appendiceal mucinous neoplasms carry a median survival of 3 years, whereas patients receiving complete CRS/HIPEC for low-grade disease achieve 70% 20-year survival (24). We recommend that healthcare institutions should develop structured protocols involving ethics committees to navigate such dilemmas, create individualized disclosure strategies that respect cultural norms while safeguarding patients’ rights to make informed treatment choices.

## 4 Conclusion

In the evaluation of patients presenting with bilateral ovarian masses and significant ascites, gynecologists typically prioritize the diagnosis of ovarian malignancy. However, it cannot be ignored that a similar clinical picture may, in rare instances, be caused by PMP secondary to a gastrointestinal mucinous neoplasm. This diagnostic possibility underscores the imperative for a meticulous preoperative assessment, a detailed history, including prior gastrointestinal surgeries, a comprehensive physical examination with careful abdominal palpation, and targeted investigations such as tumor marker assays and abdominopelvic computed tomography, must be accorded high importance. Concurrently, enhancing gynecologists’ familiarity with PMP is of paramount significance. Furthermore, although PMP is often characterized as a low-grade malignancy, it carries a high propensity for recurrence and does not preclude the possibility of advanced disease. If not diagnosed and managed promptly, patients frequently succumb to complications such as intestinal obstruction. Therefore, timely and standardized treatment, coupled with long-term follow-up, is essential. Throughout the entire diagnostic and therapeutic process, the fundamental rights and autonomy of the patient must be rigorously safeguarded.

## Data Availability

The original contributions presented in the study are included in the article/supplementary material. Further inquiries can be directed to the corresponding author.
